# Crosstalk Between the Intratumoral Microbiota and the Tumor Microenvironment: New Frontiers in Solid Tumor Progression and Treatment

**DOI:** 10.1002/cnr2.70063

**Published:** 2024-11-19

**Authors:** Qing Zhou, Lijun Zhou, Xi Chen, Qiuyan Chen, Lu Hao

**Affiliations:** ^1^ Central Laboratory The People's Hospital of Baoan Shenzhen Shenzhen China; ^2^ Department of Urology The People's Hospital of Baoan Shenzhen Shenzhen China; ^3^ Science and Education Department Shenzhen Baoan Shiyan People's Hospital Shenzhen China

**Keywords:** intratumoral microbiota, origins, progression, solid tumors, treatment

## Abstract

**Background:**

The microbiota plays a significant role in the tumor microenvironment, and its impact on tumor development and treatment outcome cannot be overlooked. Thus, it is essential to comprehend the interactions between the microbiota and the tumor microenvironment.

**Recent Findings:**

With the advent of next‐generation sequencing, microbiota research has advanced significantly in recent years. The interaction between the intratumoral microbiota and the tumor microenvironment is an emerging area of research that holds great promise for understanding and treating solid tumor progression. This crosstalk between the intratumoral microbiota and the tumor microenvironment is a complex process that involves a multitude of factors, including the immune system, cellular signaling pathways, and metabolic processes. The origin of the intratumoral microbiota differs between various solid tumor, and the quantity and diversity of intratumoral microbiota also fluctuate significantly within each solid tumor.

**Conclusion:**

The aim of this review is to provide a detailed summary of the intratumoral microbiota in various types of solid tumors. This will include an analysis of their origins, differences, and how they impact the progression of solid tumors. Furthermore, we will emphasize the significant potential that the intratumoral microbiota holds for the diagnosis and treatment of solid tumors.

## 1 Introduction

The microbiota, which is an essential component of the human body, plays a crucial role in maintaining overall health. It is mainly located in the gut and mucous membranes of the skin. Initially, it was believed that tumors were sterile until the 19th century when it was discovered that a significant number of microorganisms were present within them [1]. The development of high‐resolution electron microscopy and sequencing technologies, such as 16S rRNA, has led to advancements in microbiological and genomic studies. In solid tumors, the intratumoral microbiota is highly heterogeneous in terms of composition and abundance [2]. These intratumoral microbiota have an impact on the body's health by modulating immune, metabolic, and inflammatory pathways.

The tumor microenvironment (TME) is a complex system consisting of tumor cells, tumor‐associated fibroblasts, immune cells, inflammatory cells, adjacent mesenchymal tissue, microvasculature, and various cytokines that all play a significant role in tumor development [3, 4]. The interaction between the intratumoral microbiota and the TME is crucial for the progression of tumors [5]. Recent studies have shown that the intratumoral microbiota can have a significant impact on the TME. This impact can include the suppression of immune responses, activation of oncogenic pathways, induction of inflammatory responses, and DNA damage [6]. In turn, the local TME is altered, which can attract microbiota to the tumor site [7]. The close proximity between the intratumoral microbiota and the TME plays a crucial role in the development and metastasis of tumors [8].

In this review, we summarize in detail the origin and differences of the intratumoral microbiota in different solid tumors and the specific mechanisms by which the intratumoral microbiota and the TME interact. Furthermore, we highlight the great value of the intratumoral microbiota in the diagnosis and treatment of solid tumors.

## 2 Sources and Differences of Intratumoral Microbiota in Solid Tumors

**FIGURE 1:**
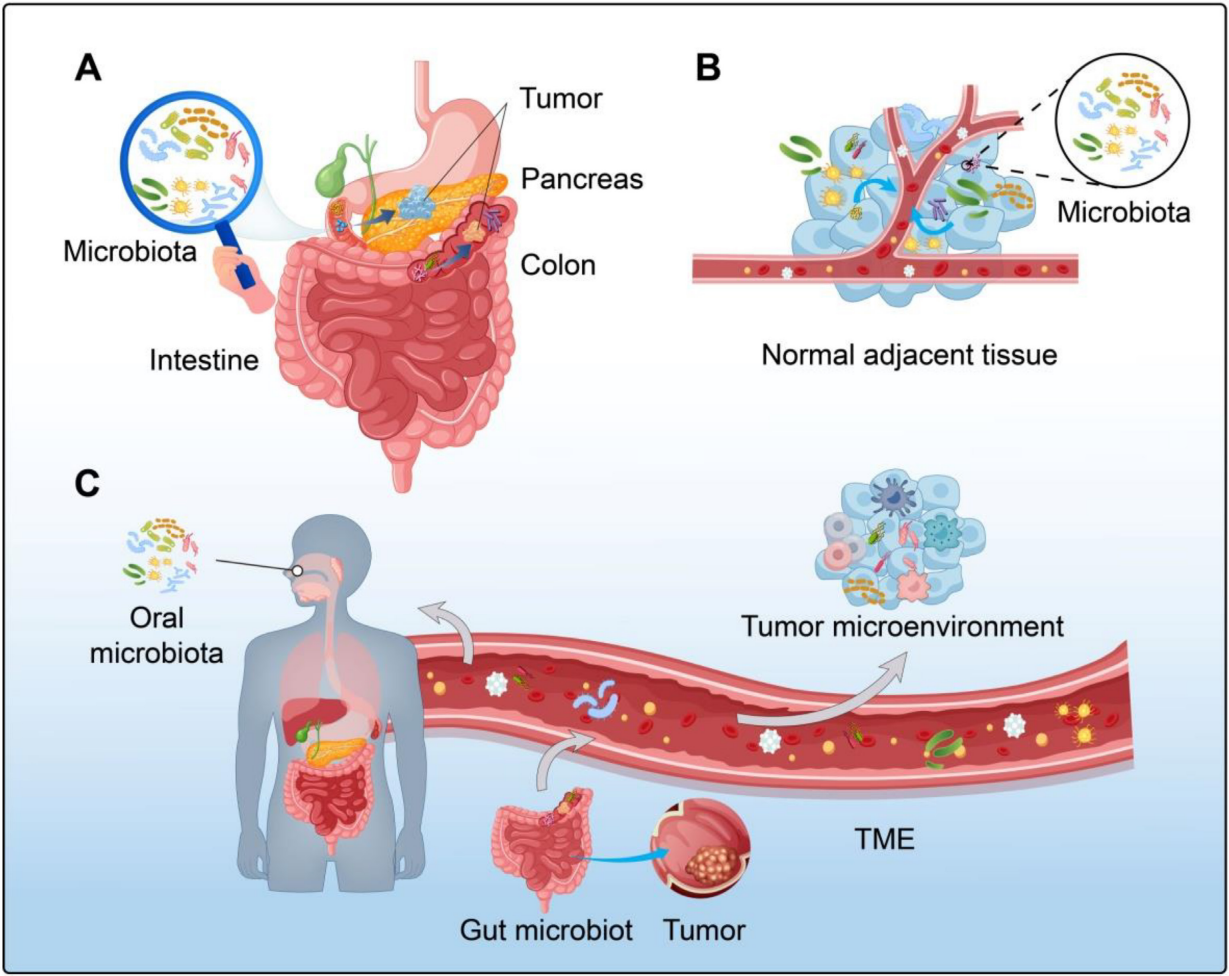
Possible explanations for the origin of intratumoral microbiota in solid tumors. (A) suggests that microbiota from oral and intestinal mucosa enter tumors by disrupting the mucosal barrier. (B) proposes that intratumoral microbiota originates from NAT, as seen in pancreatic cancer where the intratumoral microbiota is highly similar to the microbiota in the stomach, intestine, and liver. (C) suggests that microbiota can enter tumor cells through the circulatory system, as microbiota from the oral cavity and intestinal tract can reach various parts of the body through the blood circulation and invade tumor cells through damaged blood vessels.

The origin of intratumoral microbiota has been a long‐standing issue in the scientific community. According to research, there are three explanations for the potential sources of the intratumoral microbiota (Figure 1) [1, 9]. The intratumoral microbiota enter tumor cells by disrupting the mucosal barrier. Solid tumors, such as colorectal cancer (CRC), breast cancer, and lung cancer, are characterized by the exposure of organs. Microbiota that colonize the surfaces of these exposed organs can invade the tumor through the mucosal barrier. Pushalkar and colleagues have conducted studies that confirm the intratumoral microbiota originating from the gut can reach the pancreas through the pancreatic duct and destroy the mucosal barrier, thereby promoting the occurrence and development of pancreatic cancer [2, 10]. Derived from normal adjacent tissues (NAT). In 2020, Nejman et al. found that the microbiota in tumors is highly similar to adjacent tissues, suggesting that the microbiota in tumors may originate from adjacent tissues next to the tumor [2]. Intratumoral microbiota in pancreatic cancer are highly similar to microbiota in stomach, intestine, liver, and so forth [3]. Diffusion through the circulatory system and invasion into the tumor. Recent studies have shown that microorganisms such as *Fusobacterium nucleatum* (*F.n*), which originate from the oral cavity, can enter the colorectal region through the bloodstream and invade CRC cells by disrupting blood vessels [11]. In patients with CRC, *E. coli* has been found to disrupt the intestinal barrier and spread to the liver through the bloodstream. This can lead to changes in the immune microenvironment and the formation of pre‐metastatic ecological niches in the liver [12]. Tumor‐draining lymph nodes (TDLN) are located near the tumor and are responsible for draining lymphatic fluid from the tumor site. They are usually situated along the lymphatic drainage pathway of the tumor and are the first lymph nodes to encounter tumor‐released antigens. TDLN is the primary site for tumor antigen presentation to immune cells, capable of activating and expanding tumor‐specific T cells [13]. These lymph nodes enhance the immune system's recognition and attack on tumor cells, improving the effectiveness of immunotherapy. After using immune checkpoint inhibitors (ICI), lymph nodes in the body undergo remodeling. Research by Choi et al. found that following immunotherapy, certain unique subgroups of bacteria originating from the gut actively and directionally migrate to extraintestinal sites, first reaching the TDLN. There, they activate dendritic cells (DCs) and T cells, enhancing antigen presentation and anti‐tumor immune responses [14]. The advancement of next‐generation sequencing technology offers an opportunity for a detailed investigation into the origin and mechanism of intratumoral microbiota. This exploration will provide novel insights into the development and prevention of tumors in the future.

The composition of intratumoral microbiota can vary depending on the immune characteristics, oxygen levels, metabolic characteristics, and pathways of microbial origin of different solid tumors. Recent studies by Nejman and colleagues in 2020–2022 have found that each tumor contains a specific microbiota, with bacterial communities being more dominant than fungal colonies. Their research also sheds light on the spatial and population heterogeneity of intratumoral microbes in various solid tumors [15]. We summarize the different types of intratumoral microbiota found in various solid tumors (Table 1).

**TABLE 1:** Heterogeneity of intratumoral microbiota across solid tumors.

Cancer types	Genus	Status	Influence	Reference
Lung cancer	*Thermus*/*Ralstonia*	Increase	Promote tumor progression	[16, 17, 18]
	*Legionella*/*Acidovorax*	Increase		
	*Streptococcus*/*Prevotella*	Increase		
	*Aggregatibacter/Lactobacillus*	Decrease	Unclear	
Breast cancer	*Pseudomonas Bacteroides fragilis*	Increase	Promote tumorigenesis and metastasis	[19, 20, 21, 22]
	*Proteus Azomonas*	Increase		
	*Fusobacterium nucleatum*	Increase		
	*Propionbacterium/Anaerococcus*	Decrease	Unclear	
	*Caulobacter/Streptococcus*	Decrease		
Esophageal cancer	*Bacteroidetes*/*Actinobacteria*	Increase	Promote tumor progression	[23, 24, 25]
	*Fusobacterium nucleatum/Firmicutes*	Increase		
	*Campylobacter* species	Increase		
	*Proteobacteria/Bacteroidetes/Spirochaetes*	Decrease	Unclear	
Colorectal cancer	*F. nucleatum*/*Bacteroides fragilis*	Increase	Oncogenesis metastasis	[26, 27, 28]
	*Fusobacterium*	Increase		
	*Bacteroideles/Bacteroidetes*	Decrease	Unclear	
Liver cancer	*Mycobacterium avium*	Increase	Promote tumor progression	[29, 30]
	*Actinobacteria*	Increase		
	Thick‐walled bacteria	Increase	Unclear	
Stomach cancer	*Peptostreptococcus stomatis*	Increase	Promote tumorigenesis and metastasis	[31, 32, 33, 34]
	*Streptococcus anginosus*	Increase		
	*Parvimonasmicra*	Increase		
	*Slackia exigua*	Increase	Unclear	
	*Dialister pneumosintes*	Increase		
	*Bacteroidetes/Fusobacteria*	Decrease		
Pancreatic cancer	Gammaproteobacteria	Increase	Promote tumor progression	[10, 35]
	*Malassezia globos*	Increase		
	*Pseudoxanthomonas/Streptomyces*	Increase		
	*Proteobacteria/Bacteroidetes/Firmicutes*	Increase		
Prostate cancer	*Nevskia ramosa*/*Acinetobacter*	Increase	Oncogenesis metastasis	[36]
	*Pseudomonas/Escherichia*	Increase		
Ovarian cancer	*Chlamydia/Sphingomonas*	Increase	Oncogenesis metastasis	[37]
	*Acinetobacter/Brucella*	Increase		
Oral squamous cell carcinoma	*Clostridium perfringens* enterotoxin	Increase	Promote tumor progression	[38]
	*F. nucleatum and Pseudomonas aeruginosa*	Increase		[39]
Melanoma	*P. marcusii*	Unclear	Unclear	[2, 40]
	*muciniphila*		T cell activation	
	*Lachnoclostridium*			
	*Bifidobacterium*			

## 3 Crosstalk Between Intratumoral Microbiota and TME

**FIGURE 2:**
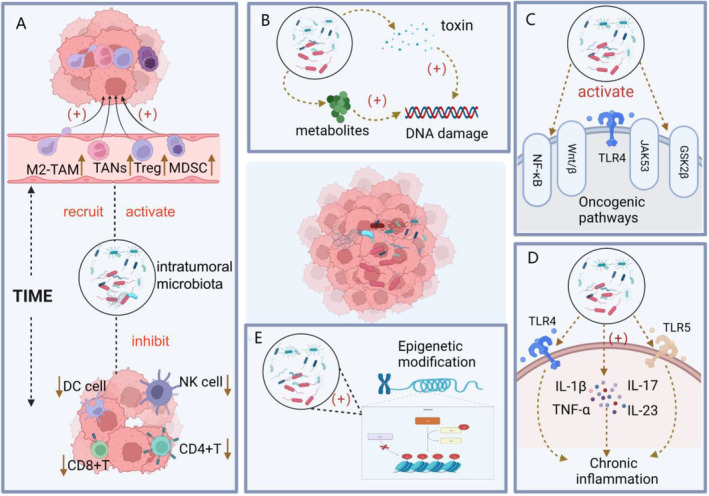
The intratumoral microbiota and TME interact to promote tumor initiation and progression, mainly involving the following five main steps. (A) Formation of an immunosuppressive microenvironment. (B) DNA damage. (C) Activation of oncogenic pathways. (D) Induction of chronic inflammation. (E) Epigenetic modifications.

The intratumoral microbiota has been shown to reshape the TME and promote tumor development. The intratumoral microbiota constructs a TME suitable for tumor development mainly through the following mechanisms: (1) formation of an immunosuppressive microenvironment, (2) DNA damage, (3) activation of oncogenic pathways, (4) induction of chronic inflammation, and (5) epigenetic modification [41] (Figure 2).

### 3.1 Formation of an Immunosuppressive Microenvironment

Tumors are known to have immunosuppressive features, which can be promoted by the microbiota through regulating innate or adaptive immune responses. Recent studies have shown that the intratumoral microbiota can form a tumor immunosuppressive microenvironment, leading to tumor growth. This is mainly achieved by promoting the activation of immunosuppressive cells such as tumor‐associated macrophage (TAM), myeloid‐derived suppressor cells (MDSCs), regulatory T cells (Tregs), and tumor‐associated neutrophils (TANs), while inhibiting the proliferation and function of anti‐tumor immune cells like T cells and NK cells. In pancreatic cancer, the bacteria target toll‐like receptors (TLRs) on macrophages, leading to TAM differentiation and recruitment of MDSCs, while inhibiting Th1 differentiation of CD4+ T cells [10]. Similarly, Borowsky et al. found that *F.n* in colon cancer recruits immunosuppressive cells such as CD11b myeloid cells, M2‐like TAMs, MDSCs, and CD103‐regulated DCs, creating a microenvironment that promotes tumor growth [42]. In oral squamous cell carcinoma, intratumoral bacterial colonization is positively correlated with the distribution of the neutrophil chemoattractant C‐X‐C motif ligand 8 (CXCL8), leading to recruitment of TANs and further suppression of the immune system. These findings suggest that targeting intratumoral bacteria may be a promising approach for cancer immunotherapy [15]. The Fap2 protein present on the surface of *F.n* in esophageal cancer binds to the inhibitory receptor T cell immunoglobulin and ITIM domain (TIGIT) on the surface of T and NK cells, leading to inhibition of their activity [43]. In lung cancer tissues, the microbiota present inside the tumor promotes the expression of cytokines such as interleukin‐1β (IL‐1β) and interleukin‐23 (IL‐23), which further promotes the proliferation of γδ T cells. However, γδ T cells release excessive amounts of interleukin‐17 (IL‐17) and interleukin‐22 (IL‐22), creating an inflammatory environment that promotes immunosuppression [18]. Furthermore, Sepich et al. discovered that the intratumoral microbiota in breast cancer produces resistance programming through pattern recognition receptor (PRR) linkage, resulting in a decreased proportion of CD8+ T cells and an increased proportion of CD4+CD25+FoxP3+ Treg among immune cells [44]. Recent studies have found that the microbiota within tumors plays a significant dual role in immune regulation. In melanoma patients, bacteria within the tumor produce HLA‐I and HLA‐II peptides, which can be presented by antigen‐presenting cells and melanoma cells. These bacterial peptides can activate the patient's immune system, particularly CD8+ and CD4+ T cells, enhancing the anti‐tumor immune response [45]. Because bacterial antigens are non‐self, they can serve as new targets for immunotherapy. Additionally, in CRC cells, the bacteria *Fusobacterium* and *Bacteroides* can enhance the anti‐tumor immune function of CD8+ T cells, inhibiting the progression of CRC [46]. The intratumoral microbiota plays a crucial role in aiding tumor cells in evading the immune system by promoting the formation of an immunosuppressive microenvironment. However, the exact mechanism by which this is achieved is yet to be fully understood and requires further exploration in future research.

### 3.2 Promotion of Deoxyribonucleotide Mutations

Genomic instability due to DNA damage is another major hallmark of tumors. Research has shown that the microbiota can cause DNA damage that leads to tumorigenesis. The intratumoral microbiota secretes metabolites, including toxins, which are instrumental in causing DNA damage [47]. Toxins, such as colibactin and lethal dilation toxin (CDT), secreted by the intratumoral microbiota have been identified as key players in inducing DNA damage. psk+ *E. coli* and Gram‐negative bacteria secrete CDT, respectively, both of which can cause cell cycle arrest and disrupt DNA self‐repair [48, 49]. Additionally, colicin secreted by pks+ *E. coli* and *Bacteroides fragilis* toxin (BFT) secreted by *Bifidobacterium fragilis* can induce the release of reactive oxygen species (ROS) and promote the secretion of IL‐17 by intestinal epithelial cells, which can directly cause DNA damage and promote colon cancer [27]. In addition to toxins, microorganisms can also promote DNA damage through the production of hydrogen sulfide and secondary bile acids. *Nucleobacteria* and *Desulfovibrio* produce hydrogen sulfide (H_2_S) which can cause DNA damage in epithelial cells and induce the expression of free radicals, promoting the development of CRC [50]. Additionally, secondary bile acids from the intratumoral microbiota can induce mitochondrial oxidative stress and promote DNA damage [51]. Although current research suggests that intratumoral microbiota and their metabolites can induce DNA damage, the specific mechanism remains unclear.

### 3.3 Activation of Oncogenes and Oncogenic Pathways

Activation of oncogenic pathways is another key factor in the development of solid tumors. Activation of oncogenic pathways such as Wnt/β‐associated protein signaling, NF‐κB pathway, and TLR pathway are critical factors in tumor progression. The intratumoral microbiota can promote tumor progression by activating these pathways [19, 52]. For instance, FadA expressed on the surface of *F. nucleatum*, which are abundant in the oral cavity, can bind to E‐calcin, leading to the disruption of epithelial homeostasis and promoting colorectal development through the activation of the Wnt/β‐catenin pathway [53]. Similarly, the *Salmonella* effector AvrA can upregulate Wnt and enhance the activity of β‐catenin, promoting the expression of Wnt/β‐catenin pathway [54]. Additionally, the BFT‐encoding gene B expressed in *B. fragilis* can activate Wnt/β‐catenin signaling and promote tumor development with the help of E‐calmodulin cleavage. The TLR4 and NF‐κB pathways have been shown to interact and work together in promoting cancer progression. Yu et al. found that *F.n* activates TLR4, which leads to the upregulation of certain micRNAs (miR‐4802, miR‐18a) and the promotion of tumor development by promoting the expression of Wnt/β‐catenin and NF‐κB signaling [55]. Additionally, the combination of Gram‐negative bacteria‐derived LPS with TLR4 can lead to the activation of the NF‐κB pathway and the expression of inflammatory factors such as IL‐6 and tumor necrosis factor α (TNF‐α), which is a key factor in generating a tumor inflammatory microenvironment and promoting tumor progression [56]. In CRC, the enrichment of *F.n* can induce the expression of the NF‐κB pathway and lead to the secretion of inflammatory cytokines, ultimately promoting the progression of the cancer. In addition to the common oncogenic pathways, the intratumoral microbiota can also activate the PI89K/ERK, JAK53, and GSK2β pathways, thereby contributing to tumor initiation and progression [57, 58]. Recent studies have found that intratumoral fungi also promote tumor progression. The fungal microbiome within tumors enhances the expression of KRASG12D in cancer cells, which in turn induces the secretion of IL‐33. The secreted IL‐33 recruits and activates TH2 and ILC2 cells in the TME. These cells stimulate tumor growth by secreting pro‐tumorigenic cytokines such as IL‐4, IL‐5, and IL‐13. Additionally, research has shown that anti‐fungal treatment can inhibit the proliferation and progression of PDAC tumors and reduce the infiltration of TH2 and ILC2 cells, thereby improving the survival rate of mice. These studies confirm that IL‐33 secretion driven by the intratumoral fungal microbiome is a critical target for PDAC progression [59]. While significant progress has been made in elucidating the mechanisms of activation of oncogenic pathways by intratumoral microbiota in relevant solid tumors such as those of the gastrointestinal tumors, further studies are needed to explore specific oncogenic mechanisms in other types of tumors.

### 3.4 Induction of Chronic Inflammation

The inflammatory microenvironment in the TME is a crucial factor that promotes tumorigenesis and progression. The intratumoral microbiota is responsible for inducing the release of inflammatory factors such as cytokines and chemokines, activating NF‐κB pathways and immune pathways, and ultimately contributing to the inflammatory microenvironment [60]. Jin et al.'s research showed that *F.n* can promote the release of IL‐1β and IL‐23, as well as the proliferation of γδ T cells. In turn, γδ T cells release large amounts of IL‐17 and IL‐22, creating an inflammatory microenvironment that promotes lung cancer progression [18]. In a mouse colon cancer model, knocking out neutrophils resulted in increased intratumoral *Akkermansia*, which in turn increased IL‐17 secretion and induced B cells to infiltrate tumors, promoting tumor progression [61]. Similarly, *F.n* recruits MDSCs and promotes the JAK–STAT signaling pathway expression, leading to the release of a large number of chemokines and interleukins, which ultimately induce an inflammatory response [62]. The intratumoral microbiota not only induces the release of inflammatory factors but also activates NF‐κΒ and TLR4 pathway, leading to the occurrence of inflammatory responses. For instance, in CRC, *F.n* can activate the NF‐κΒ pathway by binding to TLR‐4 and promote inflammatory response [63]. Similarly, microbiota on mucocutaneous surfaces can induce inflammation by activating epidermal extracellular signal‐regulated kinase‐MAP‐kinase signaling through binding to TLR‐5, which can promote the development of skin cancer [64]. Interestingly, the inflammatory microenvironment can also stimulate the proliferation of the intratumoral microbiota [65]. Peek et al. revealed that *Helicobacter pylori* secretes IL‐1β and TNF‐α in large quantities in gastric cancer, forming an inflammatory microenvironment. This environment inhibits the ability of oxyntic cells to secrete acid, which in turn promotes the proliferation of secondary bacteria [66]. The intratumoral microbiota, through the metabolites released and the inflammatory pathways involved, can effectively create a tumor inflammatory microenvironment that promotes tumor progression.

### 3.5 Involvement in Epigenetic Modification of Tumor Cells

Epigenetic modification refers to the chemical alteration of DNA and proteins on chromosomes, resulting in phenotypic changes. The mechanisms of epigenetic modifications include DNA methylation, histone modifications, and non‐coding RNAs (ncRNAs) [67]. The intratumoral microbiota and its metabolites play a significant role in the epigenetic regulation of tumors, primarily through the regulation of DNA methylation [68]. Koi et al. discovered a close relationship between enriched *F.n* in CRC tissues and CpG island methylation phenotype (GIMP) and high microsatellite instability CRC. The study found that *F.n* drives aberrant DNA methylation in CRC [69]. In a model of microsatellite unstable CRC, *Clostridium perfringens* not only positively correlated with macrophage infiltration, but also could drove CDKN2A aberrant methylation [70]. Another similar study found that the progression of chronic gastritis and gastric cancer in patients with high *H. pylori* loads was positively associated with abnormal promoter hypermethylation of genes such as DNA methyltransferase (DNMT), the Wnt inhibitor WIF1, and E‐calcineurin (CDH1) [71]. Furthermore, chronic *Fusobacteria* can induce the massive expression of 8‐oxoguanine modifications in CpG islands by producing ROS, leading to DNA methylation abnormalities [72]. Intratumoral microbiota, such as thick‐walled bacteria, *Aspergillus*, and *Bacteroidetes*, release large amounts of short chain fatty acids (SCFAs), which can cause abnormal DNA methylation and histone modification by promote or inhibit histone acetyltransferases (HATs) and deacetylases (HDACs) [73]. While the specific mechanisms of how the intratumoral microbiota affects histone modification and ncRNAs are currently unknown, investigating this further in the future is a worthwhile direction [74].

## 4 The Role of Intratumoral Microbiota in the Diagnosis and Treatment of Solid Tumors

### 4.1 Intratumoral Microbiota as a Biomarker for Cancer Diagnosis and Prognosis

The intratumoral microbiota, which varies greatly in abundance and number across solid tumors and has significant potential as a biomarker for cancer diagnosis and prognosis. In pancreatic cancer, the intratumoral microbiota originates from several sources, including the intestinal tract and oral cavity. Wei et al. conducted a study using high‐throughput 16S rRNA detection and found that the levels of *Leptozobia*, *Leptospira*, *Porphyromonas gingivalis*, and *Streptococcus* were highly enriched in the saliva of pancreatic cancer patients compared to healthy individuals, while the number of *Velociella* and *Neisseria* decreased significantly [75]. Further studies by Wei et al. confirmed these findings, revealing a significant positive correlation between the enrichment of *P. gingivalis* and the occurrence of pancreatic cancer, while the presence of mucosal *Neisseria* was associated with a low risk of pancreatic cancer. In another retrospective study on CRC, it was found that *F.n* is closely related to the microsatellite instability colon cancer phenotype and can be used as a diagnostic biomarker for cancer [76]. Furthermore, Kwong et al. found that patients enriched in *Streptococcus lysosus* or *Bacteroides fragilis* were strongly associated with a high risk of developing CRC [77]. Intratumoral microbiota such as *Bacteroides fragilis* are important as biomarkers for the diagnosis of cancer. Mouradov et al. discovered a link between gut microbiota imbalance and CRCCRC progression. By analyzing tumor and normal mucosa samples from 423 stage I to IV CRC patients using bacterial 16S rRNA gene sequencing, they identified three distinct oncomicrobial community subtypes (OCS). These subtypes were OCS1 (21%, characterized by *Fusobacterium*/oral pathogens, proteolytic activity, high‐grade tumors, MSI‐high, CIMP‐positive, CMS1, with BRAF V600E and FBXW7 mutations), OCS2 (44%, characterized by *Firmicutes*/*Bacteroidetes*, saccharolytic activity, left‐sided), and OCS3 (35%, characterized by *Escherichia*/*Pseudescherichia*/*Shigella*, fatty acid β‐oxidation, left‐sided, exhibiting CIN). OCS1 was associated with MSI‐related mutation signatures, while OCS2 and OCS3 were linked to oxidative damage. Patients with stage II/III tumors in the OCS1 and OCS3 subtypes had worse overall survival rates. This research offers a microbiota‐based CRC classification framework, aiding in better prognostication and the development of targeted microbial interventions [78].

The intratumoral microbiota can be used as a biomarker for cancer prognosis. In esophageal squamous cell carcinoma (ESCC), a high amount of *C. nucleatum* in the tumor indicates poor prognosis, low overall survival rate, and poor quality of life [79]. Similarly, a retrospective study of esophageal cancer found that high expression of *Fusobacterium* is correlated with short survival time and poor prognosis [25]. Furthermore, Chakladar et al. discovered that pancreatic cancer patients with substantial enrichment of intratumoral *Pseudomonas* and *acidophilus* (*Shigella pinnei*, *Citrobacter freundii*) had lower survival rates and poor quality of life [80]. In patients with nasopharyngeal carcinoma (NPC), the bacterial load within tumors is predominantly composed of *Corynebacterium* and *Staphylococcus*. In a cohort study by Qiao et al., it was found that patients with a high bacterial load had poorer disease‐free survival, distant metastasis‐free survival, and overall survival compared to those with a low bacterial load. The hazard ratios (HR) were 2.90, 3.18, and 3.14, respectively. The bacterial load within tumors serves as a powerful prognostic tool for NPC patients, with high levels of bacteria such as *Corynebacterium* and *Staphylococcus* being significantly associated with worse prognosis [81]. The use of intratumoral microbiota as a biomarker for cancer diagnosis and prognosis is currently advancing slowly in clinical trials. One of the key challenges is the collection of microbiota under contamination‐free conditions and ensuring high biomass. Additionally, avoiding interference with chemotherapy and antibiotics use, as well as the high cost of testing, are significant constraints to the development of this field.

### 4.2 Impact of the Intratumoral Microbiota on Chemotherapy and Immunotherapy

For malignant tumors, our current common treatment options are a combination of surgical treatment, chemotherapy, radiotherapy, and immunotherapy. The intratumor microbiota, as a key mediator that can promote tumor progression, can largely influence the efficacy of chemotherapy and immunotherapy.

#### 4.2.1 The Role of the Intratumor Microbiota in Cancer Chemoresistance

In a 2017 study, Geller et al. discovered that the intratumoral bacteria, primarily Gammaproteobacteria, metabolize gemcitabine into an inactive form called 2′,2′‐difluorodeoxyuridine with the help of the bacterial enzyme cytidine deaminase (CDD). This promotes resistance of pancreatic cancer cells to chemotherapy drugs [82]. Similarly, *F.n* was found to be highly enriched in colon cancer patients. Yu et al. observed that *F.n* can induce resistance to chemical drugs like oxaliplatin by promoting the expression of TLR4 and MYD88 signaling pathways and inducing high expression of autophagy‐related proteins ATG7 and ULK1, thereby activating the autophagy pathway [56]. *F.n* has been found to play a similar role in enhancing resistance to chemotherapy in ESCC. However, studies have shown that resistance to chemotherapeutic agents can be significantly improved when *F.n* is ablated with antibiotics [83]. This also indirectly proves that the intratumoral microbiota can directly promote tumor resistance to chemotherapy drugs.

#### 4.2.2 Impact of Intratumoral Microbiota on Immunotherapy

Cancer immunotherapy primarily involves the use of monoclonal antibodies, such as immune checkpoints (PD‐1, PD‐L1, CTLA‐4), and the identification of other ICI. The intratumoral microbiota plays a significant role in the effectiveness of ICI by influencing the immunogenicity of tumors and regulating the anti‐tumor immune response of immune cells. On the one hand, the tumor microbiota can inhibit the efficacy of immunotherapy, Pushalkar et al. discovered that pancreatic cancer patients have high levels of *Proteus*, *Bacteroidetes*, and *Firmicutes* in their intratumoral microbiota, which reduces the infiltration of CD8+ T cells, inhibits the differentiation of M1‐macrophages, and prevents the differentiation of CD1+ T cells into TH4 by activating the TLR signaling pathway. As a result, the efficacy of immunotherapy is inhibited [10]. Another study showed that in CRC patients with a large amount of *F.n* enrichment, *F.n* reaches breast cancer tissue through blood circulation, and inhibits the anti‐tumor immune response of immune cells by inhibiting the infiltration of CD8+ T cells and NK cells into the tumor tissue [20]. On the other hand: the intratumoral microbiota can enhance tumor immunogenicity and promote anti‐tumor immunotherapy. Intratumoral microbiota has been found to enhance tumor immunogenicity and promote antitumor immunotherapy, according to recent studies. Riquelme et al. discovered that intratumoral expression of *Streptomyces* and *Pseudomonas* in pancreatic cancer can activate CD4+ T cells and CD8+ T cells, thus promoting the efficacy of immunotherapy [35]. Similarly, another study found that *bifidobacteria* can enhance the efficacy of anti‐PD‐L1 and other immunotherapy by activating the antigen presentation function of DCs and promoting the infiltration of CD8+ T cells into tumor tissues [84, 85]. While the specific mechanism of how intratumoral microbiota affects chemotherapy and immunotherapy is still unclear, the rational use of antibiotics and microbiota transplantation will become a new idea for combined chemotherapy and immunotherapy for cancer treatment in the future.

## 5 Clinical Application of Intratumoral Microbiota in the Treatment of Solid Tumors

**FIGURE 3:**
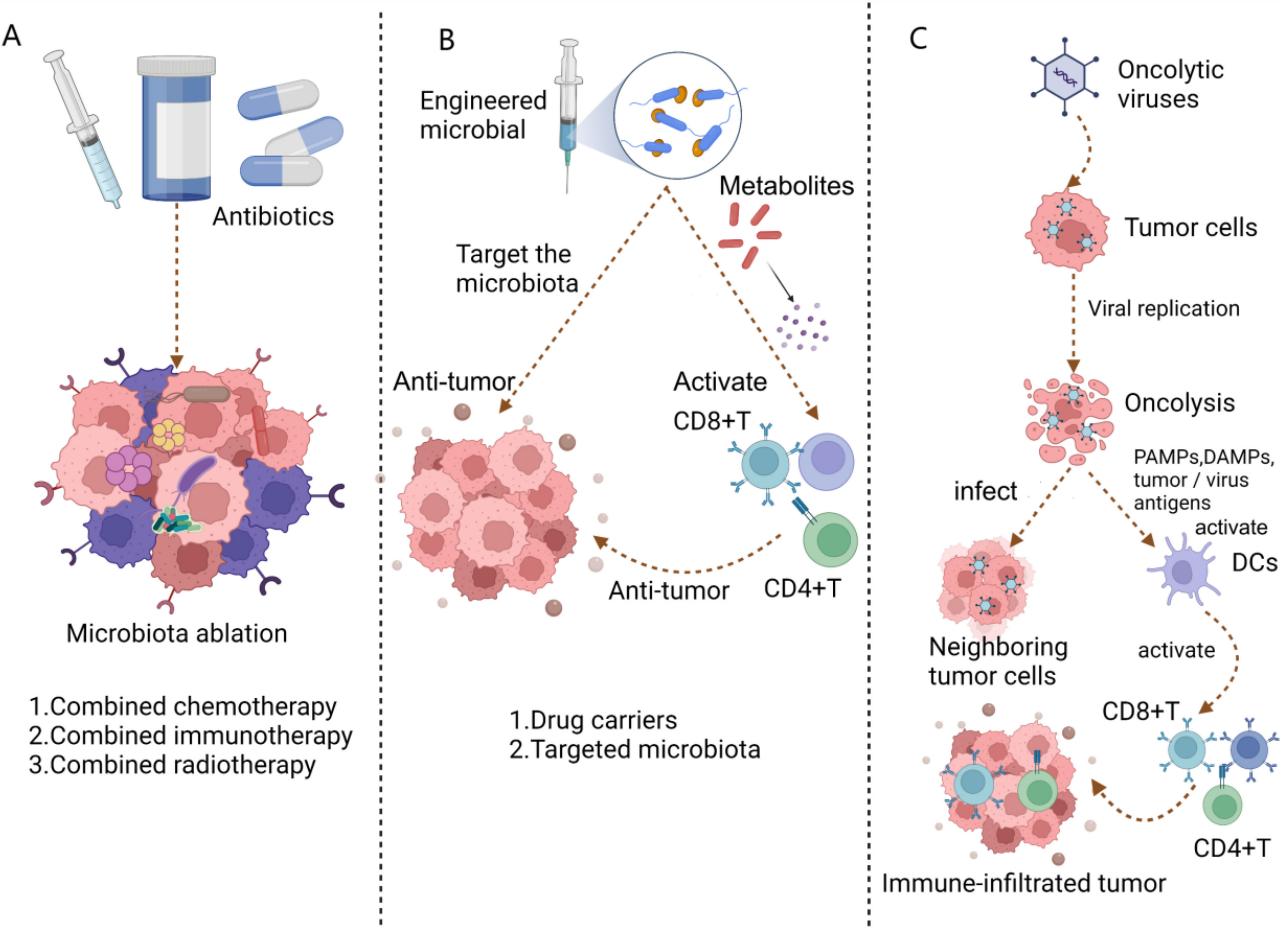
Clinical application of intratumoral microbiota in the treatment of solid tumors. (A) Microbiota ablation technology. (A) involves using antibiotics to kill harmful microbiota and improve the effectiveness of cancer treatment. (B) Engineering microbiota therapeutics. The second approach is to engineer microbiota therapeutics, which can directly target cancer cells or serve as a drug carrier for combined therapy. (C) Oncolytic virus. Utilize the technology and principle of oncolytic virus to directly or indirectly enhance the immune response for the treatment of solid tumors.

The crosstalk between the intratumoral microbiota within tumors and the TME plays a crucial role in the development of solid tumors. The clinical application of microbiota in the treatment of solid tumors remains a huge challenge for many researchers [86]. Fortunately, the advancement of microbial engineering and genetic engineering technology has led to the emergence of novel strategies for treating solid tumors. These mainly include the use of engineered microorganisms as drug carriers, oncolytic viruses and microbiota ablation technology (Figure 3).

### 5.1 Engineered Microbial Therapy

The development of synthetic biology in the 20th century has led to the use of technologies such as gene synthesis, editing, and regulation in combination with engineering to transform microorganisms in a directional manner. This has resulted in modified microbiota that can be used as drug carriers for targeted cancer treatment [87, 88]. Weber et al. discovered that certain microbiota preferentially accumulate and grow in tumor tissues with specific environments, providing a theoretical basis for the delivery of engineered microorganisms [89]. Engineered microbiota can be targeted to accumulate in the TME, for example, obligate anaerobic bacteria like clostridia can enter tumor tissue and colonize the hypoxic microenvironment of tumor tissue. Intratumoral microorganisms such as *Escherichia coli*, *Bifidobacterium*, *Listeria*, and *Salmonella* have similar mechanisms for colonizing tumor tissue and inhibiting tumor growth [90]. The use of engineered microbiota loaded with radiopharmaceuticals and chemotherapeutics holds great promise for advancing cancer treatment. Researchers have found that combining attenuated and proliferated *Listeria* with the radioactive isotope rhenium produces radioactive *Listeria* that can infiltrate pancreatic cancer tissue in large quantities, significantly inhibiting cancer progression [91]. Similarly, *Salmonella* bacteria loaded with triptolide and injected into melanoma mice have been shown to significantly reduce intratumoral angiogenesis and neutrophil infiltration, leading to tumor suppression and the formation of a hypoxic TME. In this environment, *Salmonella* can colonize the tumor in large numbers, further inhibiting tumor progression [92]. Additionally, intratumoral microbiota can serve as a carrier for gene delivery in tumor therapy. By loading Fas ligand on *Salmonella* and expressing a large amount of Fas, the bacteria can enter tumor tissue and inhibit tumor metastasis and progression in a Fas‐dependent manner [93]. In recent years, the use of probiotics by cancer patients has increased, including among those undergoing ICI treatment. Bender et al.'s research found that the interaction between the aryl hydrocarbon receptor (AhR) agonist indole‐3‐aldehyde (I3A) released by probiotics and CD8+ T cells in the TME significantly enhances antitumor immunity. The study showed that when the probiotic *Lactobacillus reuteri* (Lr) is introduced into the body, it can actively colonize within melanoma. Through the release of the dietary tryptophan metabolite I3A, it promotes the expression of CD8+ T cells and enhances the secretion of interferon‐γ, thereby boosting the effectiveness of immunotherapy. Additionally, the I3A secreted by Lr is both necessary and sufficient to drive antitumor immunity, while the loss of AhR signaling in CD8+ T cells diminishes the antitumor effects of Lr. Further research found that a tryptophan‐enriched diet can enhance the antitumor immunity induced by both Lr and ICI, depending on the AhR signaling of CD8+ T cells [94].

### 5.2 Oncolytic Viruses

Oncolytic viruses have the unique ability to infect tumor cells easily and replicate in large numbers within tumors, leading to tumor cell lysis. The release of tumor‐associated antigens during this process can be recognized by antigen‐presenting cells, which in turn activate the immune system and promote the generation of T cells and NK cells. This ultimately results in the enhancement of anti‐tumor immune function [92, 95]. In 2015, the FDA approved the use of herpes simplex virus‐1 (HSV‐1) loaded with human granulocyte‐macrophage colony‐stimulating factor (GM‐CSF) for the treatment of melanoma patients. Furthermore, studies have shown that combining oncolytic viruses with other treatment modalities can lead to even more significant therapeutic effects. The combination of oncolytic measles vaccine virus and the chemotherapeutic drug gemcitabine has been found to significantly inhibit the proliferation and progression of pancreatic cancer cells [96]. In a mouse model of neuroblastoma, the use of adenovirus Ad5 and CAR‐T in combination has shown to promote the release of chemokines RANTES and IL‐15, which attracts more CAR‐T cells to the tumor and inhibits tumor development [97]. Quoix et al. have reported that combining a modified vaccinia virus TG4010 with a PD‐1/PD‐L1 inhibitor could enhance the efficacy of immunotherapy and improve the prognosis and survival of patients with non‐small cell lung cancer [98]. We have summarized clinical trials oncolytic viruses combining with drugs such as immunotherapy or chemotherapy (Table 2).

**TABLE 2:** Clinical trials of oncolytic viruses in combination with immunotherapy or chemotherapy drugs.

NCT number	Title	Conditions	Interventions	Characteristics
NCT05644509	Study on the Treatment of Advanced Malignant Solid Tumor With Revottack and PD‐1 Inhibitor	Advanced solid tumor	Biological: Oncolytic Virus Injection#Revottack#+PD‐1	Phase 1
NCT05346484	A Study of CF33‐hNIS (VAXINIA), an Oncolytic Virus, as Monotherapy or in Combination With Pembrolizumab in Adults With Metastatic or Advanced Solid Tumors	Solid tumor Solid carcinoma Solid tumor, adult Metastatic cancer Advanced solid tumor	Biological: CF33‐hNIS Biological: Pembrolizumab	Phase 1
NCT05070221	Study of Oncolytic Virus in Combination With HX‐008 and Axitinib in Melanoma Patients With Liver Metastasis	Melanoma stage IV	Drug: Recombinant Oncolytic HSV‐2 Therapeutic Injecta (Vero Cell) for Human Use (rHSV2hGM‐CSF) Drug: Recombinant humanized anti‐PD‐ 1 monoclonal antibody for injection Drug: Axitinib	Phase 1
NCT05068453	Study of Oncolytic Virus in Combination With HX‐008 and Radiotherapy in Melanoma Patients With Liver Metastasis	Melanoma stage IV	Drug: Recombinant Oncolytic HSV‐2 Therapeutic Injecta (Vero Cell) for Human Use (rHSV2hGM‐CSF) Drug: Recombinant humanized anti‐PD‐ 1 monoclonal antibody for injection Radiation: RT	Phase 1
NCT04666688	LYT‐200 Alone and in Combination With Chemotherapy or Tislelizumab in Patients With Locally Advanced or Metastatic Solid Tumors	Metastatic cancer Solid tumor Pancreatic cancer Urothelial carcinoma Head and neck cancer Colorectal cancer	Drug: LYT‐200 Drug: Tislelizumab Drug: Gemcitabine/nab‐paclitaxel	Phase 1 Phase 2
NCT04665362	IIT Study of M1‐c6v1 Combined With SHR‐1210 and Apatinib in Patients With HCC	Advanced/metastatic hepatocellular carcinoma	Drug: Recombinant oncolytic virus M1, anti PD‐1 antibody, Apatinib	Phase 1
NCT03294486	Safety and Efficacy of the ONCOlytic VIRus Armed for Local Chemotherapy, TG6002/5‐FC, in Recurrent Glioblastoma Patients	Glioblastoma Brain cancer	Drug: Combination of TG6002 and 5‐flucytosine (5‐FC, Ancotil)	Phase 1 Phase 2
NCT03206073	A Phase I/II Study of Pexa‐Vec Oncolytic Virus in Combination With Immune Checkpoint Inhibition in Refractory Colorectal Cancer	Colorectal cancer Colorectal carcinoma Colorectal adenocarcinoma Refractory cancer Colorectal neoplasms	Drug: Durvalumab Drug: Tremelimumab Biological: Pexa‐Vec	Phase 1 Phase 2
NCT03004183	SBRT and Oncolytic Virus Therapy Before Pembrolizumab for Metastatic TNBC and NSCLC	Metastatic non‐small cell lung cancer Metastatic triple‐negative breast cancer	Biological: ADV/HSV‐tk Drug: Valacyclovir Radiation: SBRT Drug: Pembrolizumab	Phase 2

### 5.3 Microbiota Ablation Technology

Additionally, microbiota ablation technology is also a good strategy currently used in cancer treatment. For instance, Amphotericin B has been found to attenuate fungal colonization in PDAC tissues and inhibit the progression of PDAC [98, 99]. Current microbial ablation technology is limited to solving only the known harmful microbial groups that can lead to tumor development, such as HPV and *Helicobacter pylori*. It has not been extensively used for other solid tumors. However, most microbiota ablation methods rely on broad‐spectrum antibiotics to eliminate harmful microbiota, which may not always be beneficial. Numerous clinical studies and experiments have demonstrated that the use of antibiotics can hinder the efficacy of immunotherapy, including ICI, resulting in low survival rates and poor prognosis for patients [100, 101]. While it is currently feasible to engineer and genetically modify the intratumoral microbiota and combine it with chemotherapy, radiotherapy, and immunotherapy, there is a lack of comprehensive clinical studies to explore its practical value in oncology treatment. Therefore, it is crucial to investigate the clinical application of intratumoral microbiota in the treatment of solid tumors.

## 6 Limitations and Prospects

The research and application of intratumoral microbiota face multiple challenges, including limitations in detection technology, unclear mechanisms of action, safety issues in therapeutic applications, and gaps between clinical research and practical application.

(1) Challenges in Detection Technology: Accurate detection of intratumoral microbiota is a major challenge due to the typically low biomass of microbial communities in tumor samples. Additionally, the high host‐to‐bacterial DNA ratio in tumor samples can lead to biased results in amplicon‐based sequencing. Ensuring the quality and quantity of tumor samples is difficult due to the numerous steps involved in clinical sample collection. Key issues also include preventing contamination during testing and completely eliminating environmental microbial contamination. Experiments should use multiple controls to ensure accuracy. Bioinformatic analyses require customized decontamination strategies, and metagenomic data are crucial for functional interpretation. Contamination remains a persistent challenge in microbiome research, with standardization of analyses and multidisciplinary collaboration being essential solutions. Machine learning methods in the identification of intratumoral microbiota from TCGA database may be handled improperly, and these issues need validation through experimental strategies. (2) Limitations in Understanding Mechanisms of Action: Current research has not fully elucidated whether specific changes in intratumoral microbiota are causes or consequences of cancer development. The direct relationship between intratumoral microbiota and solid tumors is complex and unclear, requiring further studies to understand its mechanisms. (3) Challenges in Therapeutic Applications: Although engineered microbes have been applied in cancer treatment, their safety remains a concern [102]. Further testing in animal models and humans is needed to evaluate the safety and efficacy of these therapeutic methods. It is particularly important to study how to reduce the toxicity of these microbes and enhance their accumulation in tumor tissues to maximize their therapeutic effect [103].

The sequencing and analysis methods of intratumoral microbiota include sample collection, DNA extraction, high‐throughput sequencing, and data analysis. First, collect samples aseptically from tumor tissue, blood, or feces, and promptly freeze them to prevent DNA degradation. Next, extract high‐quality DNA using commercial kits or traditional phenol‐chloroform methods. Then, apply high‐throughput sequencing technologies such as 16S rRNA gene sequencing to analyze the taxonomy of bacteria and archaea, metagenomic sequencing to obtain the entire genomic information of the microbial community, metatranscriptomic sequencing to study gene expression, or metaproteomic sequencing to analyze proteins. Currently, 16S rRNA gene sequencing is one of the most commonly used methods for analyzing intratumoral microbiota. By amplifying and sequencing hypervariable regions (such as V4 or V3–V4 regions) of the 16S rRNA gene using the Illumina platform, microbial communities can be efficiently analyzed [104]. Open‐source analysis tools such as QIIME2 and DADA2 are used for classification and analysis. Additionally, the multiple 16S rRNA gene sequencing method developed by Nejman et al. significantly improves the coverage and resolution of bacterial species detection by amplifying five short regions in sequence along the gene (5R 16S rDNA sequencing). Despite the advantages of 16S rRNA gene sequencing in terms of low cost and accessibility, its method for determining microbial activity is limited, and sample contamination is a key concern [105]. Finally, bioinformatics tools are used to analyze the sequencing data, revealing the composition, function, and relationship of the microbiota with tumor development. Tools such as FastQC and Trimmomatic are used for quality assessment and filtering of sequencing data, removing low‐quality reads and adapter sequences. 16S rRNA gene sequencing data can be clustered and classified into operational taxonomic units (OTUs) using QIIME or Mothur; metagenomic data can be assembled using MEGAHIT or SPAdes and classified using MetaPhlAn or Kraken. Functional prediction and metabolic pathway analysis of the microbiota can be performed using tools such as PICRUSt2 or HUMAnN2.

As research progresses, an increasing number of intratumoral microbes have been found to potentially serve as prognostic indicators for various types of cancer. However, due to insufficient sample sizes, some conclusions of these studies may not be robust, highlighting the need for large multicenter cohort studies. Additionally, besides traditional chemotherapeutic mechanisms and metabolism, some studies have shown that intratumoral microbiota is related to ICI [106, 107]. Preclinical research advancements indicate the therapeutic potential of intratumoral microbiota, which may soon impact patient risk stratification, treatment choices, or clinical outcomes. Intratumoral microbiota is a crucial component of the TME, with varying origins and distributions across different solid tumors. Its presence promotes tumorigenesis and progression by fostering immune suppression, creating an inflammatory microenvironment, inducing DNA damage, and activating oncogenic pathways. Advances in spatial sequencing and engineering techniques will further clarify the origin and heterogeneous distribution of microbiota within tumors, enhancing their diagnostic and prognostic value. Understanding the specific mechanisms by which intratumoral microbiota affects solid tumor development will provide potential therapeutic ideas. Targeting intratumoral microorganisms to aggregate in tumor tissues offers new strategies for using engineered microorganisms and oncolytic viruses to treat solid tumors. Deep exploration of the complex interactions between intratumoral microbiota and the TME, utilizing different or potentially contradictory intratumoral microbiota for cancer therapy, reducing the toxicity of intratumoral microbiota and promoting their accumulation in tumor tissues, collecting high‐biomass tumor microbiota, and avoiding interference from environmental factors and antibiotics in the study of intratumoral microbiota are crucial. In the future, intratumoral microbiota will greatly improve the quality of life and survival rates of cancer patients.

## Author Contributions

**Qing Zhou:** investigation, funding acquisition, writing – original draft, conceptualization. **Lijun Zhou:** formal analysis, validation. **Xi Chen:** formal analysis, validation. **Qiuyan Chen:** validation, formal analysis. **Lu Hao:** conceptualization, supervision, writing – review and editing, project administration.

## Conflicts of Interest

The authors declare no conflicts of interest.

## Data Availability

Data sharing not applicable to this article as no datasets were generated or analysed during the current study.
